# Characterization of Selected Polymeric Membranes Used in the Separation and Recovery of Palladium-Based Catalyst Systems

**DOI:** 10.3390/membranes10080166

**Published:** 2020-07-28

**Authors:** Bongani Michael Xaba, Sekomeng Johannes Modise, Bamidele Joseph Okoli, Mzimkhulu Ephraim Monapathi, Simphiwe Nelana

**Affiliations:** 1 Chemistry Department, Faculty of Applied and Computer Sciences, Vaal University of Technology, Private Bag X021, Vanderbijlpark 1911, South Africa; BXaba@mpact.co.za (B.M.X.); joe@vut.ac.za (S.J.M.); monapathimz@gmail.com (M.E.M.); simphiwen@vut.ac.za (S.N.); 2 Department of Chemical Sciences, Faculty of Science and Technology, Bingham University, Karu PMB005, Nasarawa State, Nigeria

**Keywords:** palladium-based catalysts, polymeric membranes, separation, NF/RO membranes

## Abstract

Membrane separation processes tender a capable option for energy-demanding separation processes. Nanofiltration (NF) and reverse osmosis (RO) membranes are among the most explored, with a latent use in the chemical industry. In this study, four commercial membranes (NF90, NF270, BW30, and XLE) were investigated for their applicability based on the key structural performance characteristics in the recycling of Pd-based catalysts from Heck coupling post-reaction mixture. Pure water and organic solvent permeabilities, uncharged solute permeability, swelling, and catalyst rejection studies of the membranes were conducted as well as the morphological characterization using Fourier transform infrared, field emission gun scanning electron microscopy, and atomic force microscopy. Characterization results showed trends consistent with the manufactures’ specifications. Pure water and organic solvent fluxes generally followed the trend NF270 > NF90 > BW30 > XLE, with the solvent choice playing a major role in the separation process. Pd(PPh_3_)_2_Cl_2_ was well rejected by almost all membranes in 2-propanol; however, XLE rejects Pd(OAc)_2_ better at high pressure in acetonitrile. Our study, therefore, revealed that the separation and reuse of the two catalysts by NF90 at 10 bar resulted in 97% and 49% product yields with 52% and 10% catalyst retention for Pd(OAc)_2_ while Pd(PPh_3_)_2_Cl_2._ gave 87% and 6% yields with 58% and 36% catalyst retention in the first and second cycles, respectively. Considering, the influence of membrane–solute interactions in Pd-catalyst rejection, a careful selection of the polymeric membrane and solvent, a satisfactory separation, and recovery can be achieved.

## 1. Introduction

Separation technology has evolved during the 20th century, driven primarily by advances in the petroleum industry. Numerous technologies such as distillation, extraction, and adsorption have been universally used [1]. The use of membranes in nonaqueous media has drawn a lot of attention in recent years owing to their inferior energy demands and ease of use [2]. A promising field, particularly in pressure-driven processes, is organic solvent nanofiltration (OSN) [3]. Lab-scale and commercial-scale applications of OSN membranes have been reported [4,5]. In the advance and application of the membrane process, characterization of modelling and optimization are essential steps [6,7]. A consistent and reliable method of measuring the separation performance of membranes is essential and allows end-users to make an informed selection [8]. Membrane characterization parameters may be described as either performance related or morphology related. Performance-related parameters describe membrane functionality such as flux, rejection, and molecular weight cutoff (MWCO). Morphological parameters which include physical and chemical parameters describe the structure of the membrane [9]. Important morphological parameters include porosity and roughness of the membrane.

Molecular weight cutoff is described as the molecular weight for which 90% rejection of the solute is achieved by the membrane [10]. The MWCO concept is based on the observation that molecules generally get larger as their masses increase. As molecules get larger, sieving effects due to steric hindrance increase and a larger molecule is rejected by the membrane more than the smaller molecule. MWCO may also be related to diffusion since larger molecules diffuse more slowly than smaller molecules [11]. Hilal et al. [12] suggested that MWCO determination depends on experimental conditions such as the nature of the feed solution and the type of membrane module. They showed this by using a mixture of polyethyleneglycols (PEG) with different molecular weights. Their results also showed that some membranes have larger and smaller pores respectively than specified by the manufacturer.

Porosity has been regarded as another useful parameter to describe separation in membranes. Porosity is usually expressed as pore size, density, pore size distribution (PSD), or the effective number of pores in the membrane’s upper active layer [13]. Roy, Sharqawy, and Lienhard [14] have theoretically probed the effects of log-normal pore size distribution on the rejection of uncharged solutes and NaCl. They showed that the theoretical log-normal function is not apposite for nanofiltration (NF) membranes due to the large pore size tail of the distribution dictating rejection and flux. Their results also showed that elucidation based on uncharged solute data alone cannot give functional quantitative information about the membrane pore size distributions. However, when used in conjunction with other surface characterization techniques, they showed good agreement in pore size distribution.

Košutić et al. [15] investigated the porosity of NF and reverse osmosis (RO) membranes by permeation of uncharged compact organic molecules. To ascertain the influence of the porous structure of the membrane skin on the retention mechanism of different solutes, the PSDs were determined at almost 690 kPa. Their results showed a distinction between the PSD of NF and RO membranes. The RO membranes revealed a wide PSD and bimodal distribution, with maxima at 0.52 and 0.80 nm. The PSD of NF membranes exhibited maxima at larger pore sizes, the first one between 0.95 and 1.10 nm and the second maximum around 1.55 nm. This was clear evidence of the existence of larger surface pores.

Interest in the application of membrane technology in homogeneous catalyst separation was evident in the period from 2000 to 2002. The effective application of coated polymeric ultrafiltration membrane for the photocatalytic degradation by organic pharmaceuticals [16] and the cationic phenothiazine dye remediation using optimized polyelectrolyte assisted ultrafiltration [17] was some of the reported areas of interest. Other studies used solvent-resistant NF (SRNF) membranes. Of these, the polymer-based membranes suggest persuasive prospects for SRNF with unparalleled cost-efficiency [18]. Vandezande, Gevers, and Vankelecom [10], however, highlighted the dilemma of comparing retention data from different studies, as these are application specific. The different properties of solvents, membranes, filtration mode, and operating conditions affect the results in membrane separations.

The early applications of OSN technology were involved in the recovery and reuse of the high-value palladium (Pd) catalyst in Heck, Sonogashira, and Suzuki reactions [19,20]. These processes recorded major successes in the removal of about 95% of residual Pd and separation of the product from the catalyst and ionic liquid after Suzuki coupling reactions [21,22]. However, the size of this catalyst is the same as the product and may result in poor separation. Further, the premium utilization of Pd is as pharmaceutical chemicals and active pharmaceutical ingredients (APIs) and the daily permitted oral exposure to Pd in a pharmaceutical ingredient of less than 10 mg of Pd per kilo of API (<10 ppm) [23]. The premium applications of palladium in synthetic biology as an in vivo catalyst [24], the pharmaceutical industry, and electrocatalysis [25] have become a subject of interest to most researchers in recent years. Hence, palladium was dubbed the king of transition-metal catalysts [26].

In light of this, it was of interest to investigate the separation characteristics of common polymeric membranes in the recovery of palladium (Pd) catalysts in aqueous and organic media with an attempt to separate and reuse the catalysts from Heck coupling post-reaction mixture. The membranes were characterized for pure water permeability, pure solvent permeability, surface morphology, chemical structure, and uncharged solute rejection measurements. The separation performance of different membranes in different solvents was studied. This work will serve as an archetype for evaluating NF/RO membranes’ performance in the recovery and reuse of palladium (Pd) catalysts. The data will help identify the suitable combination of membranes and solvents for use in order to achieve effective palladium (Pd) catalyst separation and recycling. It will also shed some light on the key performance-related or morphology-related parameters responsible for effective catalyst separation and recycle.

## 2. Experimental

### 2.1. Instrumentation

The determination of sugar and alcohol concentrations was achieved by the use of Lambda 25 UV-VIS spectrometer (Perkin-Elmer, Waltham, MA, USA) and Clarus 500 Gas Chromatograph (Perkin-Elmer, Bridgeport Avenue, Shelton, USA), respectively. Membrane characterisation was done on the following instruments: Field Emission Gun Scanning Electron Microscopy (FEG-SEM) (Carl Zeiss SMT GmbH, Peabody, MA, USA), Atomic force microscopy (AFM) (Park Scientific Instruments, Janderstrasse, Mannheim, Germany), and Spectrum 400 Fourier-transform infrared Spectrophotometer (FTIR) fitted with a universal attenuated total reflection (ATR) sampler (Perkin-Elmer, Waltham, MA, USA)

### 2.2. Chemicals Reagents

Five solvents were chosen for the study. These are acetonitrile, methanol, ethanol, and 2-propanol, all analytical reagent grade, and water. These solvents are commonly used in organic synthesis and were chosen because of their solvating properties. The solvents also represent the two classes of solvents, those which coordinate via oxygen and those coordinating via selective donor atoms such as nitrogen, known as oxic and anoxic solvents, respectively. Water and 2-propanol were used for membrane characterization. Acetonitrile and 2-propanol were used in catalyst separation studies. The physical properties of the respective solvents are given in Table S1 of the Supporting Information [27]. 

**Figure 1:**
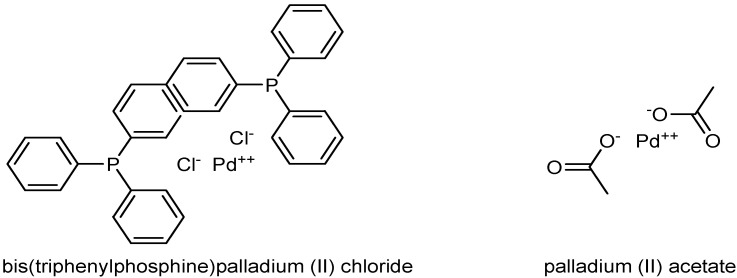
Structures of the catalysts used.

Uncharged solutes, glucose and sucrose, were also selected for the determination of molecular weight cutoff of the membranes. These solutes were supplied by Merck. Two transition-metal catalysts were used in the study, namely palladium (II) acetate complex of molecular weight 224 g mol^−1^ and bis(triphenylphosphine)palladium (II) chloride complex (Sigma-Aldrich) of molecular weight 701.91 g mol^−1^ (Figure 1). 

### 2.3. Membranes

Four commercially available NF/RO membranes were used for this study (NF90, NF270, BW30, and XLE). These were thin-film composite membranes of various characteristics supplied by Dow/FilmTec (Minneapolis, MN, USA) (Table S2 of the Supporting Information). 

### 2.4. Analytical Procedure

**Figure 2:**
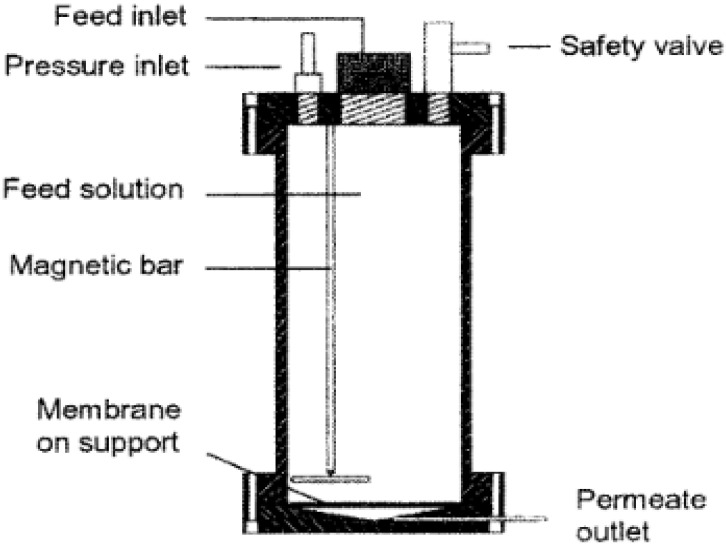
Dead-end filtration unit used for retention measurements [28].

A bench-scale stainless steel dead-end module with a capacity of 1.2 litres was operated at pressures of 25 bar with nitrogen gas was used (Figure 2). 

The unit was fitted with a Teflon-coated magnetic stirrer supported on the upper lid by a steel rod. Stirring was required to homogenize the sample and to minimize concentration polarization [28]. Disc samples of the different membranes with a diameter of 9 cm and an effective area of 0.0064 m^2^ were cut and placed on a porous support disc. The holdup volume underneath the porous support disc was ~1 mL. The permeate was collected from a Teflon tube into a measuring cylinder. Filtration measurements were performed by loading feed solutions with a volume ranging from 250–600 mL at 24 °C. The first 20 mL of permeate collected was discarded. Thereafter, 10 mL of permeate was collected at a specified time. The flux was obtained by Equation (1):

(1) $$J=\frac{V}{A\cdot t}$$

where *V* is the volume of permeate, *A* is the membrane area, and *t* is the time. 

### 2.5. Membrane Swelling Experiment

The interaction of the solvents with the membrane physical structure was further investigated by measuring the swelling tendency of the membranes. Membranes were cut and dried at room temperature in an open dish. Each dried membrane was weighed and immersed in the selected solvents. After an equilibrium time of approximately 30 minutes, the membrane was removed from the solvent and quickly dried with a soft tissue to remove the solvent from the external surface before weighing. Swelling of the membrane was calculated by Equation (2) [29]:

(2) $$Q=\frac{1}{\rho_{s}}\frac{W_{wet}-W_{dry}}{W_{dry}}$$

In the equation, *Q* is the swelling, *W_wet_* is the mass of wet membrane, *W_dry_* is the mass of dry membrane, and *ρ_s_* is the density of the solvent.

### 2.6. Membrane Characterization

Feed solutions of uncharged solutes were prepared with concentrations of 0.1 vol % for the alcohol and 0.1 wt% for the sugars. The concentrations of the feed and permeate (sugars and alcohols) were estimated by the Anthrone method [30] and gas chromatography, respectively. Characterization of the surface morphology and chemical structure of the polymer gave information on the specific chemistry and orientation of the structure of the functional groups present in the membrane active layer [31].

### 2.7. Catalyst Rejection

Dissolution of catalysts was done in various solvents (Table 1) and then filtered at 10 and 20 bar. The concentration and rejection coefficient of the catalyst in the permeate and feed solutions were determined by UV-VIS spectroscopy and Equation (3), respectively:

(3) $$R=\left(1-\frac{C_{\mathrm{permeate}}}{C_{\mathrm{feed}}}\right)100$$

where *C*_permeate_ and *C*_feed_ are concentrations of the catalyst in the permeate and feed, respectively.

### 2.8. Catalyst Separation and Reuse

**Figure 3:**
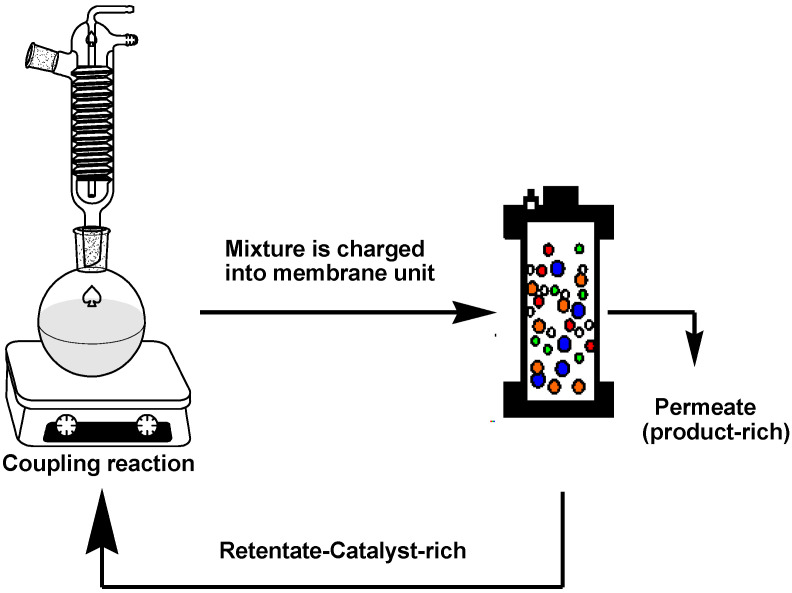
Schematic of catalyst separation and reuse procedure.

The rejection of the catalysts in a Heck coupling reaction mixture was investigated based on the hypothesis that sufficient catalyst rejection will enable separation of the catalyst from the mixture, however, keeping in mind that the catalyst rejection behaviour in a multicomponent solution will be expected to be different from that of the single- and binary-component solutions. The coupling reaction was performed as described by Nair et al. [32] with slight modification and was allowed to proceed for 4 to 6 h. At this point, the reaction was stopped, cooled to room temperature, and immediately charged into the dead-end unit for filtration (Figure 3). 

A feed sample was taken for UV-VIS analysis before filtration, after which filtration was performed at 10 bar until ~70% of the volume had permeated. The retentate was also sampled for UV-VIS analysis, and catalyst rejection by the membranes was calculated according to Equation (3). This concentrated retentate solution was then transferred back to the reaction flask. Fresh reactants and solvent were topped up for a second reaction run. The filtration protocol and reaction run were repeated for several cycles until no further change in conversions could be observed. This procedure was repeated two times per membrane to get an overall concept of the efficiency of catalyst separation.

### 2.9. Data Analysis

Data analysis was performed using OriginPro 2015 Sr 1 b9.2.257, and comparisons between different membranes were carried out using a one-way analysis of variance (ANOVA). Data were expressed as mean ± SD of triplicate determinations. Significant was considered at *p* < 0.05.

## 3. Results

### 3.1. Pure Water Permeability

Measuring the membrane’s dependence on pressure, it is possible to characterize the porosity of the membrane’s active layer [33]. Pure water permeability was investigated by using the Kedem–Katchalsky model for irreversible thermodynamics [34]. According to the model, the relationship between pure water flux and pressure is expressed in Equation (4).

(4) $$J_{w}=A_{w}\left(∆P-\sigma \cdot ∆\pi \right)$$

where *J_w_* is the water flux, *A_w_* is the membrane permeability, Δ*P* is the pressure difference, *σ* is the reflection coefficient, and Δ*π* is the osmotic pressure difference. In the case where only pure water is present, the osmotic pressure difference becomes zero; therefore, Equation (4) is reduced to Equation (5):

(5) $$J_{w}=A_{w}\cdot ∆P$$

**Figure 4:**
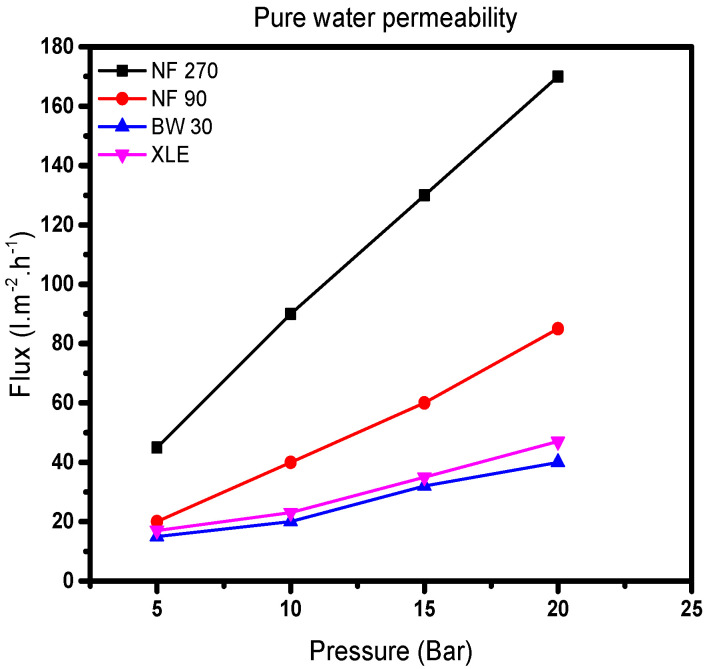
Plot of water flux (*J_w_*) against pressure difference (Δ*P*) of the membranes.

The results show a linear relationship between water flux and applied pressure (Figure 4). The water flux through all the membranes shows an increase with increasing pressure. Values of *A_w_,* obtained from the slope of the model, show that NF270 has the largest pure water permeability followed by NF90, XLE, and BW30. It was noted that the pure water permeability value of NF270 is twice that of NF90 and more than four times that of XLE and BW30 (Table 1).

Consequently, it is expected that the pore size distribution would follow the same trend. From Equation (3), the effective membrane pore radius will increase proportionally with pure water permeability. This is in line with the literature; NF90 and NF270 are classified as “tight” and “loose” membranes, respectively [35]. The results show that BW30 and XLE are similar in terms of pore sizes; however, Nghiem and Coleman [36] proposed the absence of pores in BW30. Presumptuously, XLE has the same nonporous structure as BW30, which is also in line with the low-pressure RO membrane (LPRO) supplier’s classification [37]. NF90 has a pure water permeability which lies between that of the NF270 and the RO membranes. Therefore, it is expected that NF90 will behave in a similar way to the RO membranes. Besides, the pure water permeability results agree to some extent (Table S2 of the Supporting Information).

Permeate flow results at supplier’s standard test conditions show that NF90 and XLE have the lowest permeate flow followed by BW30 and NF270. However, when considering the maximum flow through these membranes, NF90 has the lowest maximum flow followed by BW30, XLE, and NF270. This conflicting observation points to changes in pore structure with increasing flow through each membrane; by implication, there is a similarity in the properties of NF90 and RO membranes (BW30 and XLE). NF270 has the largest pore size with the highest flow and pure water permeability values.

### 3.2. Organic Solvent Permeability

**Figure 5:**
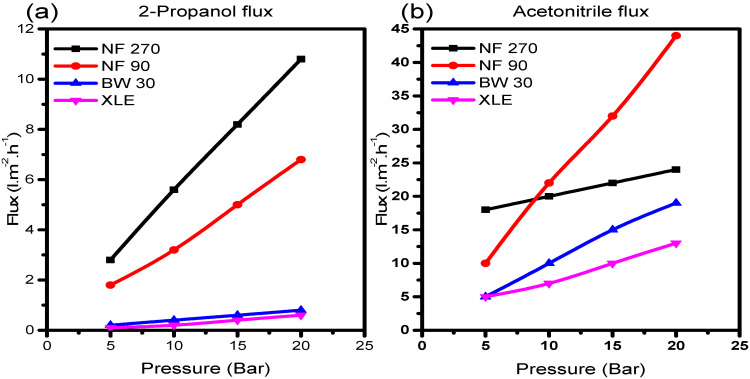
(**a**) 2-Propanol and (**b**) Acetonitrile fluxes through the membranes showing pressure dependence.

The Hagen–Poisseuille equation in Equation (6) explains the relationship between flux, pressure, and viscosity, where an increase in pressure results in a corresponding increase in flux. Hence, solvent flux for viscous flow is described by Equation (6) [38].

(6) $$J_{s}=\left(\frac{\varepsilon \cdot r^{2}}{8\cdot ∆x\cdot r}\right)\left(\frac{∆P}{\eta }\right)$$

where *J_S_* is the solvent flux, *ε* is the porosity, *r* is the average pore radius, Δ*P* is the pressure difference, *η* is the viscosity, Δ*x* is the effective membrane thickness, and *τ* is the tortuosity factor. Figure 5a,b shows the plots of solvent flux vs. pressure for 2-propanol and acetonitrile, respectively.

There was a good correlation with Equation (5); all solvents showed steady constant fluxes which increased with increasing pressure. Also, each solvent exposed to NF270 generally yielded higher fluxes than NF90, BW30, and XLE, with a trend almost similar to the observation in pure water permeability measurements. On the contrary, BW30 with the lowest pure water permeability coefficient gave higher fluxes compared to XLE. This phenomenon indicates the variation in membrane behaviour in the presence of a different solvent. Therefore, the resultant rejection behaviour of the membranes will similarly be perturbed.

The same phenomenon was observed with acetonitrile; strangely with NF90, the trend of the initial solvent fluxes appeared to be similar to what was observed in the flux of 2-propanol. However, as the pressure increases, sudden changes in pore structure become evident in NF90. The solvent fluxes suddenly increase to become the highest of all the membranes; an indication of NF90 pore structure alteration due to the interaction with acetonitrile molecules. The size of the solvent molecule has an effect on the morphology of the polymer at a molecular level [39], causing the polymer chains to either relax or contract as the solvent molecules penetrate the matrix.

**Figure 6:**
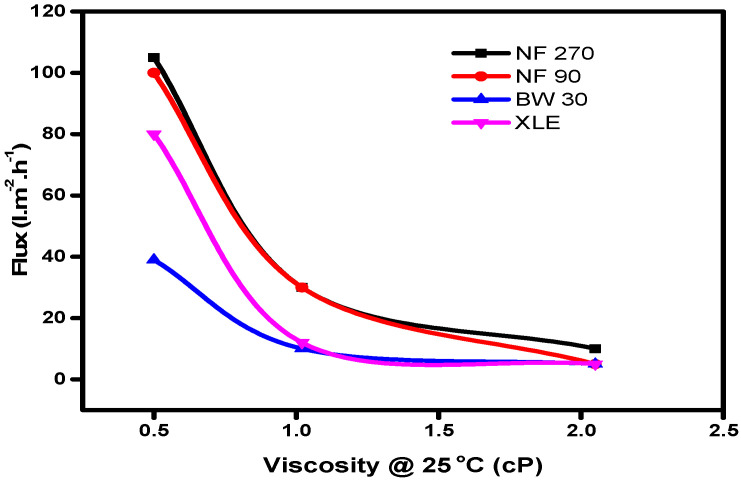
Graphs showing the relationship between flux and viscosity.

Equation (5) highlights the influence of viscous flow on solvent transport in nanofiltration membranes, which is evident from the plot of solvent flux against viscosity (Figure 6).

The results showed an increase in solvent flux with decreasing viscosity of solvents; hence, a solvent with low viscosity will flow through the membrane with more ease than a solvent with high viscosity. The resistance to flow will, therefore, lead to lower fluxes in the membranes, which collaborates the Hagen–Poisseuille model.

The effect of ease of flow is in line with the resistance-in-series model developed by Machado, Hasson, and Semiat [40] and described by Equation (7). The model describes the permeation of the solvent through composite polymeric membranes. It has been reported that permeation through the membrane pores will only occur when the applied pressure overcomes the surface energy difference [38].

(7) $$J=\frac{∆P}{\phi^{I}\left[∆\gamma +f_{1}\cdot \mu \right]+f_{2}\cdot \mu }$$

where *f*_1_ and *f*_2_ are constants characterizing the individual mass transfer coefficients and pore radii, ϕ is the solvent parameter, and Δ*γ* is the surface energy difference between the membrane and solvent.

Yang, Livingston, and Freitas Dos Santos [41] have highlighted the difficulty of relating viscosity and surface tension of solvents to their flux. In their work, they included water as a solvent and showed that water has a higher viscosity and surface tension than methanol but higher flux in all the membranes tested. They concluded that the difference in the solvent fluxes through the membranes cannot be explained solely by differences in viscosity and surface tension. Interactions between the membrane and solvent, dependent on the membrane material and properties, also have to be taken into account. In the current study, our results show some correlation between the viscosity and flux of the selected solvent and therefore agree with the observation of Yang et al.

### 3.3. Membrane Swelling Experiment

Solvent causes swelling of the polymeric material caused by solvent contact, leading to a negative effect on the separation efficacy and lifetime of the polymeric membrane material [42]. The three solvents used in coupling reactions and catalyst retention studies and the resultant effect are presented in Table 2.

Tarleton et al. [43] observed that the degree of the swelling has a significant effect on flux than the viscosity of the mixture. Further, Silva, Han, and Livingston [5] ruled out swelling as a justification for higher flux in ethylacetate compared to toluene when in contact with STARMEM 122 and MPF50 membranes. Hence, the solvent may lead to pore-structure changes such as swelling of the polymer matrix. In our study, the swelling behaviour of the membranes in acetonitrile, 2-propanol, and water was investigated.

The results show that all the membranes swell more in the organic solvent than in water. Swelling is more pronounced in 2-propanol, followed by acetonitrile and lastly water. The results are in agreement with the observations of Zhao and Yuan [44].

**Figure 7:**
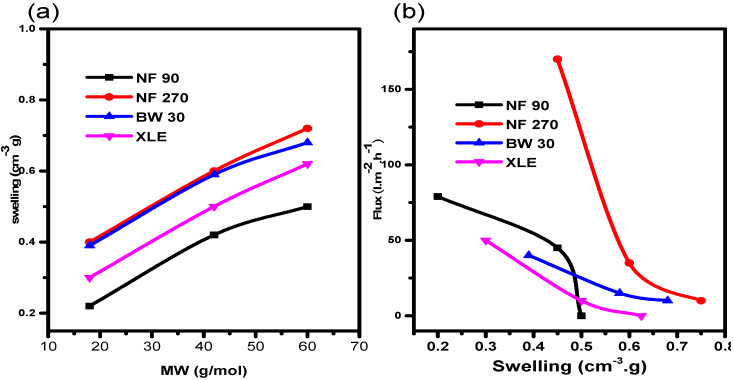
Relationship between membrane (**a**) swelling and molecular weight and (**b**) swelling and flux.

It was important to rationalize our observations of higher swelling in organic solvents compared to water. To begin with, the relationship between swelling and molecular weight of the solvent was investigated (Figure 7a). The results show that, for the selected solvents, membrane swelling increases linearly with increasing molecular weight. The results show that swelling is more pronounced in 2-propanol (highest molecular weight). It can be seen that NF270 showed higher swelling in all the solvents followed by BW30, XLE, and NF90, in that order. In light of the observations above, the influence of swelling on solvent flux was investigated and it reveals that the flux declines with an increasing swelling degree (Figure 7b). NF270 shows the steepest flux decline. A strange observation from the results is that of NF90. The membrane also shows a rather steep flux decline despite low swelling degree when compared to NF270, BW30, and XLE. A steady flux decline with increasing swelling was observed for BW30 and XLE. This observation may be due to the close solubility parameter of the solvent with the NF90 causing chain shrink [45].

It is clear from the results that swelling has a negative effect on the flux behaviour of the membranes, which is attributed to changes in the polymer matrix, as documented by other authors [5,46,47]. Freger et al. [48] explained that swelling leads to disruption of cross-linking and formation of new hydrophobic and hydrophilic functional groups in the membrane structure. This change in cross-linking is common in NF membranes [39,49]. The cross-linking chains expand during swelling, therefore, increasing the size of the pores. This results in a decrease in the flux of components through the membrane.

The opposite has also been cited as a possible mechanism of swelling in NF membranes [50]. In this process, the polymer chains shrink, therefore, increasing the size of the pores. This leads to an increase in solvent fluxes and a reduction in the rejection of solutes of interest. Our results agree with the former process, pointing to decreased pore sizes with swelling.

Miller-Chou and Koenig [39] have based the differences in dissolution behaviour to mass and momentum transport on the swelling polymer matrix. They concluded that the nature of the polymer and the differences in rigidity are the main parameters that determine polymer swelling behaviour. For the membranes studied, it is clear that there are similarities in structure and dissolution behaviour as well as how the selected solvent impacts the membranes. These membrane-solvent interactions however do not alter the membrane structures drastically. For catalyst separation to be effective, the membrane-solvent interactions should not be detrimental. It was therefore concluded that the solvents selected are fit for later use in catalyst retention and separation studies.

### 3.4. Scanning Electron Microscopy (SEM) and Atomic Force Microscopy (AFM)

**Figure 8:**
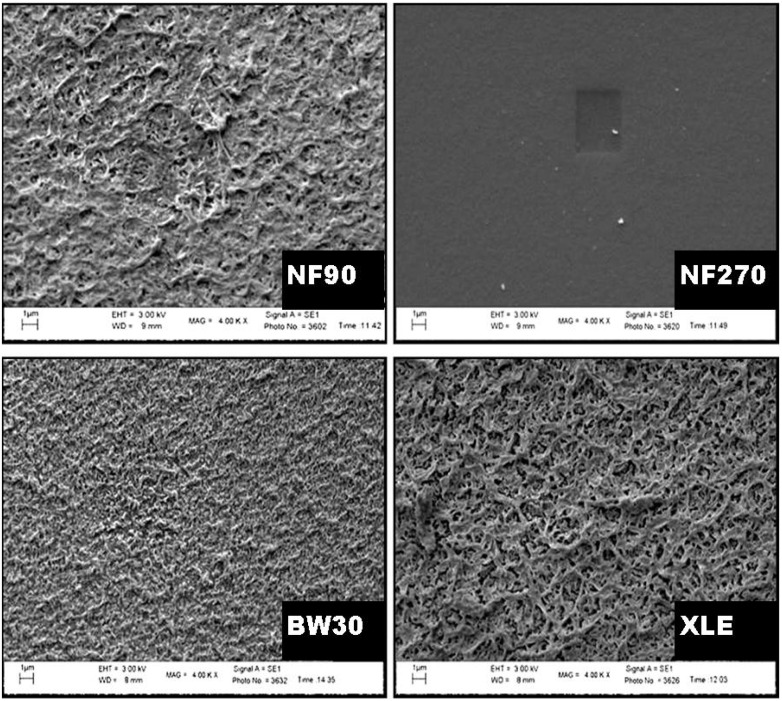
Surface morphology of NF90, NF270, BW30, and XLE.

The SEM micrograph of the surface showed the texture and appearance of the membranes (Figure 8), with a homogeneous surface covering. NF90, BW30, and XLE showed a similar type of appearance and texture, while NF270 showed some exceptions with a fairly smooth surface and texture. NF90 and XLE are the most identical membranes in terms of surface roughness. However, BW30 also showed some degree of roughness surface but with smaller “hills” and “valleys” compared to NF90 and XLE. From the micrograph, one can conclude that “tight” NF membranes are generally rougher compared to “loose” NF membranes.

The images of the microstructure of the membranes show that they consist of three characteristic layers (Figure S1 of the Supporting Information). These are labelled as (i) top barrier layer, (ii) porous polysulfone layer, and (iii) non-woven polyester support layer [51]. Each layer has a definite function. The top layer serves as a separation barrier, separating components based on the MWCO [52]. The polysulfone layer acts as a support layer designed to withstand high pressures during filtration. The non-woven support adds structural support to the whole composite membrane. This layer is tailored to generate a hard, smooth, and compact surface [53].

The rough surface of the NF90 membrane was further characterized by AFM (Figure S2 of the Supporting Information), and the observations are in agreement with the SEM result (Table 3).

Long Duc Nghiem, Coleman, and Espendiller [54] studied the surfaces of NF90 and NF270 and noted the difference in surface roughness between the two membranes. NF90 had a mean roughness of 69.9 nm, while NF270 had a roughness of 5.5 nm. The texture of the membranes may influence fouling tendency. Membranes with a rougher texture are more prone to colloidal fouling than smooth membranes. Yang and Craig [55] explained that preferential clogging of the “valleys” on the surface of the membrane occurs, resulting in flux decline.

### 3.5. Uncharged Solute Permeability

Uncharged solutes were used to determine the molecular weight cutoff of the membranes. Bellona et al. [11] have highlighted that this definition may be vague and can vary between 60 and 90% depending on the manufacturer’s protocols. The concept of MWCO is based on the observation that, as molecules get larger, separation by sieving due to steric hindrance increases. For uncharged solutes, the convective flow of the solute through the membrane is not influenced by electrostatic interactions. Therefore, a larger molecule will be rejected more than a smaller molecule. Diffusion may also play a role in the MWCO, since a larger molecule may diffuse more slowly than the smaller molecule.

In the current study, three solutes were used to determine the MWCO, propanol (*MW* = 60 g mol^−1^); glucose (*MW* = 180 g mol^−1^), and sucrose (*MW* = 342 g mol^−1^) (Table S3 of the Supporting Information). The trend in terms of the MWCO is NF270 > NF90 > BW30 > XLE and highlights the influence of membrane–solute interactions. It can be seen from the results that the larger solute is rejected more than the smaller one. According to the steric hindrance pore model, the larger solute will experience more frictional resistance during diffusive and convective transport through the membrane more than the smaller solute.

### 3.6. FTIR Structural Determination

The chemical structure of the membrane was probed to understand the behaviour of the membranes. According to the patent by Cadotte, Dow FilmTec membranes are prepared by interfacial polymerization [56]. This polymerization process is based on the combination of 1,3-phenylene diamine and triacid chloride of benzene. The resulting membrane is usually referred to as fully aromatic [54]. In the case where piperazine is used, instead of 1,3-phenylene diamine, the resulting membrane is referred to as semi-aromatic [57]. The FTIR result showed that NF90, BW30, and XLE fit the characteristics of fully aromatic polyamides while NF270 is a semi-aromatic polyamide (Table S4 of the Supporting Information).

The fully aromatic membranes show a common band at ~1663 cm^−1^. This band can be assigned to C=O stretching. The band is referred to as amide band I for aromatic polyamides. The membranes also share common bands at ~1543 cm^−1^. This band is assigned to in-plane N–H bending and C–N stretching. The band is also known as amide band II and is characteristic of aromatic polyamides [58]. It can be seen from the results that amide bands I and II are missing from the spectrum of NF270. This is an indication of a semi-aromatic structure. All the membranes however do share common bands. These are observed in polyamides in general. The band at 1585 cm^−1^ can be assigned to the C=C bond stretching in aromatic rings. The strong band at ~1238 cm^−1^ present in all the membranes spectra can be assigned to C–O stretching. This band points to the presence of carboxylic acid. It has been mentioned that the presence of these carboxylic acids leads to a chemically resistant and robust polymer. The FTIR results show that NF90, BW30, and XLE are similar in terms of chemical structure. NF270 is somewhat different due to the aliphatic influence in its chemical structure.

Overall, the results confirm the presence of different functional groups in the polymeric membranes. The presence of functional groups in the active layer determines the physicochemical properties of the membrane. McGilvery et al. [59] have stated that the type and concentration of functional groups present in the membrane active layer affect membrane–solute and membrane–solvent interactions. In turn, membrane performance such as permeability and rejection is influenced. In the membranes studied, it is clear that differences in chemical structure have an impact on membrane performance. In light of this, a preliminary investigation into the rejection of two catalysts by the membranes was performed.

### 3.7. Catalyst Rejection

Catalyst rejection measurements in acetonitrile and 2-propanol were performed; the two solvents have different properties (Table S1 of the Supplementary information). Therefore, it can be expected that their dielectric constant and molecular size will be different. In light of these characteristics, it was of interest to compare catalyst retention in the said solvents.

#### 3.7.1. Rejection in Acetonitrile

**Figure 9:**
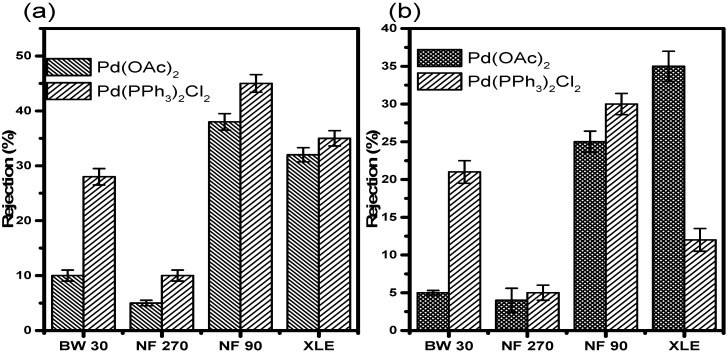
Catalyst rejection in acetonitrile at (**a**) 10 and (**b**) 20 bar.

Retention results in acetonitrile at 10 and 20 bar (Figure 9) showed very low retention of Pd(OAc)_2_ in all the membranes irrespective of the pressure. At 10 bar, NF90 showed the highest retention of 40 ± 1.5%. XLE showed the second-highest retention of 36 ± 1.3%. BW30 showed a mere 13 ± 1.0% retention, while NF270 was the least with less than 10 ± 0.5% of the catalyst retained. The membranes showed similar retention of Pd(PPh_3_)_2_Cl_2_ in acetonitrile. NF90 once again showed the highest retention of 48 ± 1.6%. XLE showed retention of 38 ± 1.4%. BW30 showed improved retention of 30 ± 1.5%. NF270 also showed increased retention of 12 ± 1.0%. The increase in retention is however still insignificant with much of the catalyst still permeating through the membranes. The trend with respect to the rejection of Pd(PPh_3_)_2_Cl_2_ is NF90 > XLE > BW30 > NF270.

The retention measurements at 20 bar show lower retention of the catalysts compared to measurements at 10 bar. In this instance, XLE showed the highest Pd(OAc)_2_ retention of 38 ± 2.0%. NF90 showed retention of 28 ± 1.4%. This observation points towards a 30% decrease in the retention when compared to measurements at 10 bar. BW30 and NF270 showed similar retention, less than 10%. The retention in BW30 also showed a 45 ± 0.3% decrease when compared to measurements at 10 bar. The trend relating to Pd(OAc)_2_ retention at 20 bar is XLE > NF90 > BW30, NF270.

The retention of Pd(PPh_3_)_2_Cl_2_ in acetonitrile at 20 bar was also lower in all the membranes when compared to 10 bar measurements. NF90 showed the highest retention of 32 ± 1.4%. A strange observation was that of BW30. The membrane showed retention of 24 ± 1.5% which is higher than that in XLE. The latter showed retention of 15 ± 1.5%. There is almost a 33% difference in the retention between the two membranes. In acetonitrile, the rejection of Pd (PPh_3_)_2_Cl_2_ is lower than Pd (OAc)_2_ in XLE membranes; this may be due to increased membrane fouling and concentration polarization at 20 bar compared to 10 bar. The results, therefore, indicate that membrane characteristics and pressure do significantly influence retention in acetonitrile.

#### 3.7.2. Rejection in 2-Propanol

**Figure 10:**
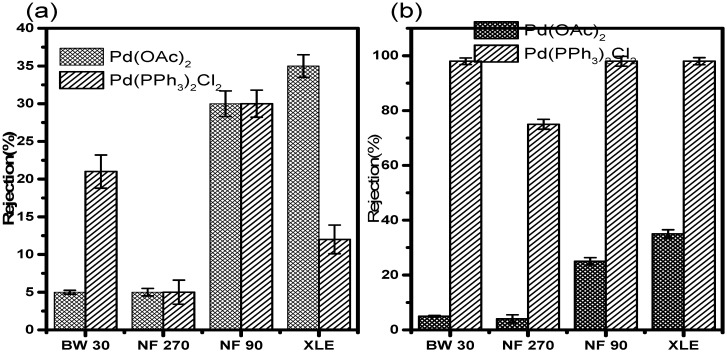
Catalyst rejection in 2-propanol at (**a**) 10 and (**b**) 20 bar.

Retention results in 2-propanol at 10 and 20 bar (Figure 10) showed slightly higher retentions compared to those in acetonitrile. At 10 bar, NF90 showed the highest Pd(OAc)_2_ retention of 74 ± 1.8%. XLE showed retention of 44 ± 1.5%. NF270 and BW30 showed poor retention of 9 ± 0.5% and 4 ± 0.3%, respectively. The Pd(OAc)_2_ rejection trend at this point is NF90 > XLE > NF270 > BW30. The most obvious observation from the results is the retention of Pd(PPh_3_)_2_Cl_2_. It can be seen that the catalyst was well retained by all membranes. NF90, BW30, and XLE showed retentions of >99%. NF270 showed retention of 86%, which is still fairly high when compared to retention measurements in acetonitrile.

The results at 20 bar show a decrease in catalyst retention. XLE showed the highest retention with 38 ± 1.5% of Pd(OAc)_2_ retained. NF90 showed retention of 33 ± 1.4%. This is an indication of a 55% reduction in retention compared to 10 bar measurements. NF270 and BW30 showed very poor retention of 8 ± 1.5% and 6 ± 1.3%, respectively. The rejection trend at this point is XLE > NF90 > NF270 > BW30. The retention of Pd(PPh_3_)_2_Cl_2_ at 20 bar did not change much. Most of the membranes showed very good retention of the catalyst. NF90, BW30, and XLE showed retentions up to >99%. NF270 showed retention of 78 ± 1.8%. The results, therefore, indicate that pressure does not significantly influence retention in 2-propanol.

#### 3.7.3. Rejection in Water

Retention measurements were performed in water to determine the separation of the catalysts (Pd (OAc)_2_ and PdCl_2_) from aqueous media. However, the low solubility of Pd (OAc)_2_ in water should be kept in mind. PdCl_2_ was dissolved in a small amount of HCl before dilution with distilled water to achieve the total dissolution of the complex.

**Figure 11:**
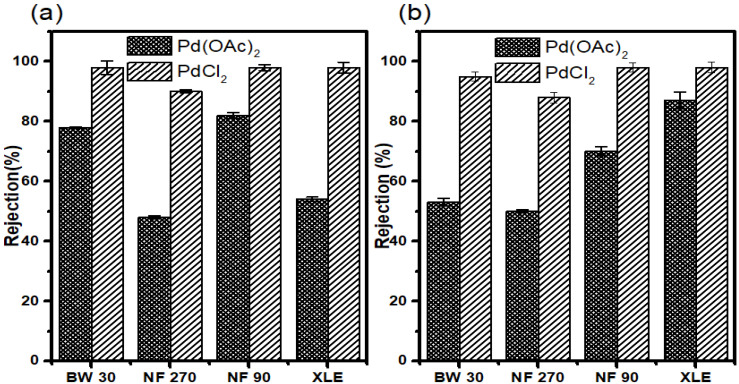
Catalyst rejection in the water at (**a**) 10 and (**b**) 20 bar.

The results show good retention of Pd (OAc)_2_ in all membranes. NF90 showed the highest retention of 84 ± 1.3%; however, BW30 showed comparable retention of 81 ± 1.5%. XLE and NF270 showed reasonable retention of 66% and 50%, respectively. The trend observed for Pd (OAc)_2_ retention in water is different from that observed in retention measurements where organic solvents were used. In the former, the trend is NF90 > BW30 > XLE > NF270 (Figure 11a).

All membranes showed very good retention for PdCl_2_ with NF90, BW30, and XLE showing retentions of up to >99%. NF270 also showed notable retention of 92 ± 0.8%. These retention results are similar to those of Pd (PPh_3_)_2_Cl_2_ in 2-propanol.

The results may be strange; however, a small MW of PdCl_2_ plays a vital role compared to the smaller MW of Pd(OAc)_2_. It was expected that PdCl2 would be more poorly retained than Pd(OAc)_2_, but this was not the case. The results, therefore, highlight that other transport mechanisms have to be taken into consideration when addressing retention data.

The results show that 70 ± 1.7% of Pd(OAc)_2_ was retained in NF90. XLE showed an increase in retention compared to measurements at 10 bar, with 69 ± 0.5% retention achieved. BW30 showed a 31 ± 0.5% reduction in the retention compared to 10 bar measurements. The membrane showed retention of 56 ± 2.1%. NF270 realized a slight increase in retention with 53 ± 1.5% retention. The rejection trend at this point is NF90 > XLE > BW30 > NF270. Overall, the membranes showed the highest catalyst retention in water compared to retention in organic solvents (Figure 11b).

The catalyst retention results in acetonitrile, 2-propanol, and water show the influence of solvent–solute interactions. Solvents differ in the way they interact with solutes. The concept of solvation is of importance in addressing solvent–solute interactions. Solvation has been defined as the phenomenon in which each dissolved molecule or ion is surrounded by a shell of solvent molecules [60].

Reichardt and Welton [61] have explained that solvation increases with increasing polarity of the solvent. Our results show that catalyst retention generally increases with increasing polarity of solvent (Table S1 of the Supporting Information). It can be assumed then that better solvation of the solute leads to increased retention. This is in line with observations by Jeroen Geens et al. [62]. They observed higher retentions in methanol than in ethanol. They based their results on the solvation properties of the two solvents.

Membrane–solute interactions are also of importance; the overall retention results indicates that the solute size and steric hindrance effects are critical factors. A large-size catalyst was rejected more, on average, irrespective of the solvent as a result of membrane–solute interactions in which parameters such as surface resistance and mass transfer resistance have influence.

### 3.8. Catalyst Separation and Reuse

The concept of catalyst separation was investigated in the Heck coupling post-reaction mixture. The separation of two catalysts (Pd(OAc)_2_ and Pd(PPh_3_)_2_Cl_2_) by NF90 at 10 bar was studied. The reaction results of Pd(OAc)_2_ from reaction mixture run 18, and Pd(PPh_3_)_2_Cl_2_ from run 26 are listed in Table 4.

**Figure 12:**
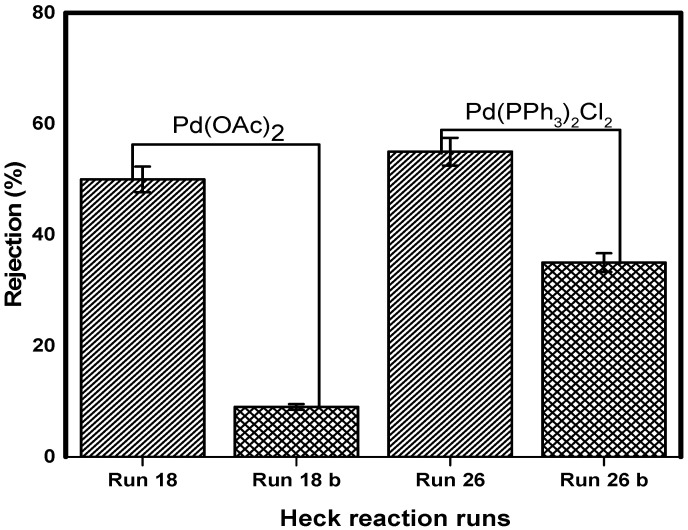
Heck reaction catalyst retention and recycle in acetonitrile at 10 bar.

The coupling reaction–recycle procedure was run for two cycles for each catalyst and the retention results of Pd (OAc)_2_ (Figure 12). The reaction of run 26 performed with Pd (PPh_3_)_2_Cl_2_ reached reasonable conversions after 4 h, with 87% yields obtained. The first filtration cycle resulted in a 58% retention of the catalyst (Figure 12).

The retentate from this cycle was used to initiate the reaction of run 26b with fresh reactants and solvent. This reaction was plagued by palladium black formation.

An insignificant reaction yield of 6% was realized after 8 h. For totality, the second filtration cycle was performed. This cycle yielded 36% catalyst retention. It is not clear whether the precipitated palladium black had influenced catalyst retention. The retentate from this cycle contained a deactivated catalyst. This was evident in the reaction of run 26c, which did not show any conversion after 8 h. It was decided that the catalyst had lost activity.

These results highlight that catalyst decomposition has to be taken into account to address losses during nanofiltration of transition-metal catalysts. It has been shown that catalysts are reduced from Pd(II) to Pd(0) during the Heck reaction [63,64]. This Pd (0) complex is prone to deactivation into palladium black entities [65,66]. The exact mechanism of deactivation is beyond the scope of this study. It should be kept in mind that our system was not isolated, and therefore, deactivation due to oxidation was imminent.

The results however show that catalyst recycling is possible. This procedure appears to be a trade-off between reduced reaction rates and catalyst loss. Our system did not show the robustness, and only two nanofiltration cycles were possible. This is somewhat unreasonable when compared to other author’s reports of up to ten filtration cycles [67,68]. Our observations show that many factors have to be taken into account when considering such a catalyst-recycling process.

## 4. Discussion

There is a decrease in retention with increasing pressure in all the membranes (Figure 9 and Figure 10), which is in contrast to the observations by Scarpello et al. [69] that observed increased retentions with increasing pressure; however, their study involved the use of solvent-resistant polyimide and polysiloxane membranes. Hence, the chemical properties will differ from the polyamide membranes used in our study. Consequently, membrane performances such as flux and rejection will also differ.

The catalyst retention results in acetonitrile and 2-propanol show the influence of solvent–solute interactions. The concept of solvation is of importance in addressing solvent–solute interactions [60]. Reichardt and Welton [61] have explained that solvation increases with increasing polarity of the solvent. Our investigation of acetonitrile and 2-propanol showed that better catalyst retention was observed in the latter, contradicting the influence of solvents. It was expected that better solvation of the solute should lead to increased retention, as reported by Jeroen Geens et al. [62]. They observed higher retentions in methanol than in ethanol. They based their results on the solvation properties of the two solvents. This solute–solvent behaviour shows that there are other interactions involved in membrane separation.

Membrane–solute interactions are also of importance. Looking at the overall retention results, it can be seen that solute size and steric hindrance effects come into play. The larger catalyst was rejected better on average irrespective of the solvent. This observation points towards membrane–solute interactions in which parameters such as surface resistance and mass transfer resistance have influence. The larger catalyst will experience more of these effects than the smaller catalysts. Separation is achieved by size exclusion. Membrane–solvent interactions have also shown an effect. NF90 showed an increase in pore sizes and therefore higher fluxes in acetonitrile. This was different from the behaviour in 2-propanol. All the interactions discussed above highlight the complexity of membrane separation in organic media.

## 5. Conclusions

The main goal of applying membrane process in the potential separation and recovery of palladium-based catalyst systems from reaction mixtures was achieved through a series of structured activities which include a review of literature, characterization of the membrane, catalyst retention, and recycling. Good membrane stability was achieved in the selected organic solvents. Also, the catalyst retention revealed a good degree of membrane–solute interaction (steric hindrance and size exclusion). The larger catalyst was rejected more by all the membranes irrespective of the solvent used; however, the smaller catalyst was the most poorly rejected. The XLE membrane was found to reject Pd(OAc)_2_ better at high pressure when dissolved in acetonitrile; however, Pd(PPh_3_)_2_Cl_2_ was well rejected by almost all membranes when dissolved in 2-propanol instead of acetonitrile. Besides, the degree of catalyst-separation was found to be influenced by membrane–solvent, solute–solvent, and membrane-solute interactions. However, these issues are currently not yet fully understood and provide exciting challenges for further research. In the meantime, these issues are major limitations for the industrial application of the membrane–catalyst separation protocol.

It can be concluded that the integrity of NF90, NF270, and BW30 membranes are not affected by solvent types and pressure between 10–20 bar. On the contrary, the separation integrity of XLE in acetonitrile is compromised at a pressure of 20 bar. For an effective separation, recovery, and reuse of selected the Pd-based catalysts, a more elaborate investigation involving the application of the response surface (statistical) in the optimization of the dead-end filtration unit must be conducted.

## Figures and Tables

**Table 1:** Pure water permeability of the membranes.

Membrane	*A_w_* (ℓ·m^−2^·h^−1^·bar^−1^)
NF90	3.8
NF270	8.9
BW30	2.1
XLE	2.4

**Table 2:** Results of swelling measurements.

Solvent	Swelling (cm^−3^ g)			
	NF90	NF270	BW30	XLE
Water	0.23	0.41	0.38	0.31
Acetonitrile	0.43	0.60	0.58	0.51
2-Propanol	0.50	0.72	0.68	0.63

**Table 3:** Atomic force microscopy (AFM) roughness measurements.

Membrane	Roughness (nm)
NF90	124.99
NF270	11.40
BW30	95.52
XLE	135.60

**Table 4:** Heck catalyst reaction–recycle procedure.

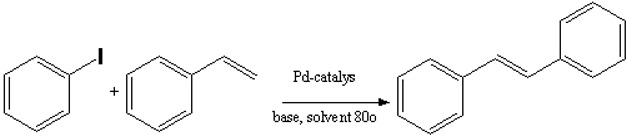						
Run	Catalyst	Base	Ligands	Solvent	Yield	Catalyst Retention
18	Pd(OAc)2	Et3N	PPh3	Acetonitrile	97%	52%
18b	Pd(OAc)2	Et3N	Bipy	Acetonitrile	49%	10%
18c	Pd(OAc)2	Et3N	Bipy	Acetonitrile	No reaction	
26	Pd(PPh3)2Cl2	Et3N	PPh3	Acetonitrile	87%	58%
26b	Pd(PPh3)2Cl2	Et3N	PPh3	Acetonitrile	6%	36%
26c	Pd(PPh3)2Cl2	Et3N	PPh3	Acetonitrile	No reaction	

## Supplementary Materials

The following are available online at https://www.mdpi.com/2077-0375/10/8/166/s1, Supplementary materials from this study which include AFM image and SEM micrographs are provided as supporting information.

## References

[1] Noble R.D., Agrawal R. (2005). Separation need for the 21st century. Ind. Eng. Chem. Res..

[2] Geens J., Boussu K., Vandecasteele C., Van der Bruggen B. (2006). Modelling of solute transport in non-aqueous nanofiltration. J. Membr. Sci..

[3] Boam A., Nozari A. (2006). Fine chemical: OSN—A lower energy alternative. Filtr. Sep..

[4] Yamada T., Kobayashi Y., Ito N., Ichikawa T., Park K., Kunishima K., Ueda S., Mizuno M., Adachi T., Sawama Y. (2019). Polyethyleneimine-Modified Polymer as an Efficient Palladium Scavenger and Effective Catalyst Support for a Functional Heterogeneous Palladium Catalyst. ACS Omega.

[5] Silva P., Han S., Livingston A.G. (2005). Solvent transport in organic solvent nanofiltration membranes. J. Membr. Sci..

[6] Bargeman G., Vollenbroek J.M., Straatsma J., Schroën C.G.P.H., Boom R.M. Nanofiltration of Multi-Component Feeds. Interactions between Neutral and Charged Components and Their Effect on Retention. https://research.utwente.nl/en/publications/nanofiltration-of-multi-component-feeds-interactions-between-neut.

[7] Sarkar S., Sondhi K., Das R., Chakraborty S., Choi H., Bhattacharjee C. (2015). Development of a mathematical model to predict different parameters during pharmaceutical wastewater treatment using TiO2 coated membrane. Ecotoxicol. Environ. Saf..

[8] Radjabian M., Abetz V. (2020). Advanced porous polymer membranes from self-assembling block copolymers. Prog. Polym. Sci..

[9] Merlet R.B., Pizzoccaro-Zilamy M.A., Nijmeijer A., Winnubst L. (2020). Hybrid ceramic membranes for organic solvent nanofiltration: State-of-the-art and challenges. J. Memb. Sci..

[10] Vandezande P., Gevers L.E.M., Vankelecom I.F.J. (2008). Solvent resistant nanofiltration: Separating on a molecular level. Chem. Soc. Rev..

[11] Bellona C., Drewes J.E., Xu P., Amy G. (2004). Factors affecting the rejection of organic solutes during NF/RO treatment—A literature review. Water Res..

[12] Hilal N., Al-Abri M., Al-Hinai H., Abu-Arabi M. Characterization and Retention of NF Membranes Using PEG, HS and Polyelectrolytes. https://squ.pure.elsevier.com/en/publications/characterization-and-retention-of-nf-membranes-using-peg-hs-and-p.

[13] Mulder M. (1996). Basic Principles of Membrane Technology.

[14] Roy Y., Sharqawy M.H., Lienhard J.H. (2015). Modeling of flat-sheet and spiral-wound nanofiltration configurations and its application in seawater nanofiltration. J. Membr. Sci..

[15] Košutić K., Furač L., Sipos L., Kunst B. Removal of Arsenic and Pesticides from Drinking Water by Nanofiltration Membranes. https://www.researchgate.net/publication/223653466_Removal_of_arsenic_and_pesticides_from_drinking_water_by_nanofiltration_membranes.

[16] Chakraborty S., Loutatidou S., Palmisano G., Kujawa J., Mavukkandy M.O., Al-Gharabli S., Curcio E., Arafat H.A. (2017). Photocatalytic hollow fiber membranes for the degradation of pharmaceutical compounds in wastewater. J. Environ. Chem. Eng..

[17] Dasgupta J., Singh A., Kumar S., Sikder J., Chakraborty S., Curcio S., Arafat H.A. (2016). Poly (sodium-4-styrenesulfonate) assisted ultrafiltration for methylene blue dye removal from simulated wastewater: Optimization using response surface methodology. J. Environ. Chem. Eng..

[18] Lim S.K., Goh K., Bae T.H., Wang R. (2017). Polymer-based membranes for solvent-resistant nanofiltration: A review. Chin. J. Chem. Eng..

[19] Garrett C.E., Prasad K. (2004). The art of meeting palladium specifications in active pharmaceutical ingredients produced by Pd-catalyzed reactions. Adv. Synth. Catal..

[20] Molnár Á. (2011). Efficient, selective, and recyclable palladium catalysts in carbon-carbon coupling reactions. Chem. Rev..

[21] Han S., Wong H.T., Livingston A.G. (2005). Application of organic solvent nanofiltration to separation of ionic liquids and products from ionic liquid mediated reactions. Chem. Eng. Res. Des..

[22] Wong H.T., See-Toh Y.H., Ferreira F.C., Crook R., Livingston A.G. (2006). Organic solvent nanofiltration in asymmetric hydrogenation: Enhancement of enantioselectivity and catalyst stability by ionic liquids. Chem. Commun..

[23] European Medicines Agency (2016). Guideline on the Specification Limits for Residues of Metal. Catalysts or Metal. Reagents.

[24] Miller M.A., Askevold B., Mikula H., Kohler R.H., Pirovich D., Weissleder R. (2017). Nano-palladium is a cellular catalyst for in vivo chemistry. Nat. Commun..

[25] Tsuji J. Palladium Reagents and Catalysts: New Perspectives for the 21st Century. https://books.google.com.ng/books?id=RDT0OUdlj0MC&pg=PA90&redir_esc=y&hl=en.

[26] Ritter S.K. (2016). A user’s guide for palladium acetate. C&EN Glob. Enterp..

[27] Steve Murov Properties of Solvents Used in Organic Chemistry. http://murov.info/orgsolvents.htm#TABLE.

[28] Krieg H.M., Modise S.J., Keizer K., Neomagus H.W.J.P. (2005). Salt rejection in nanofiltration for single and binary salt mixtures in view of sulphate removal. Desalination.

[29] Noordman T.R., Wesselingh J.A. (2002). Transport of large molecules through membranes with narrow pores—The Maxwell-Stefan description combined with hydrodynamic theory. J. Membr. Sci..

[30] Málnási-Csizmadia A. (2013). Introduction to Practical Biochemistry.

[31] Xu P., Drewes J.E. (2006). Viability of nanofiltration and ultra-low pressure reverse osmosis membranes for multi-beneficial use of methane produced water. Sep. Purif. Technol..

[32] Nair D., Luthra S.S., Scarpello J.T., White L.S., Freitas dos Santos L.M., Livingston A.G. (2002). Homogeneous catalyst separation and re-use through nanofiltration of organic solvents. Desalination.

[33] Qasim M., Badrelzaman M., Darwish N.N., Darwish N.A., Hilal N. (2019). Reverse osmosis desalination: A state-of-the-art review. Desalination.

[34] Katchalsky A., Kedem O. (1962). Thermodynamics of Flow Processes in Biological Systems. Biophys. J..

[35] Micari M., Diamantidou D., Heijman B., Moser M., Haidari A., Spanjers H., Bertsch V. (2020). Experimental and theoretical characterization of commercial nanofiltration membranes for the treatment of ion exchange spent regenerant. J. Membr. Sci..

[36] Nghiem L.D., Coleman P.J. (2008). NF/RO filtration of the hydrophobic ionogenic compound triclosan: Transport mechanisms and the influence of membrane fouling. Sep. Purif. Technol..

[37] (2012). DOW FILMTEC^TM^ Reverse Osmosis Membranes Technical Manual. www.dowwaterandprocess.com.

[38] Robinson J.P., Tarleton E.S., Millington C.R., Nijmeijer A. (2004). Solvent flux through dense polymeric nanofiltration membranes. J. Memb. Sci..

[39] Miller-Chou B.A., Koenig J.L. (2003). A review of polymer dissolution. Prog. Polym. Sci..

[40] Machado D.R., Hasson D., Semiat R. (2000). Effect of solvent properties on permeate flow through nanofiltration membranes. Part II. Transport model. J. Membr. Sci..

[41] Yang X.J., Livingston A.G., Freitas Dos Santos L. (2001). Experimental observations of nanofiltration with organic solvents. J. Membr. Sci..

[42] Ebert K., Koll J., Dijkstra M.F.J., Eggers M. (2006). Fundamental studies on the performance of a hydrophobic solvent stable membrane in non-aqueous solutions. J. Membr. Sci..

[43] Tarleton E.S., Robinson J.P., Millington C.R., Nijmeijer A., Taylor M.L. (2006). The influence of polarity on flux and rejection behaviour in solvent resistant nanofiltration-Experimental observations. J. Membr. Sci..

[44] Zhao Y., Yuan Q. (2006). Effect of membrane pretreatment on performance of solvent resistant nanofiltration membranes in methanol solutions. J. Membr. Sci..

[45] Ben Soltane H., Roizard D., Favre E. (2013). Effect of pressure on the swelling and fluxes of dense PDMS membranes in nanofiltration: An experimental study. J. Membr. Sci..

[46] Babu B.R., Rastogi N.K., Raghavarao K.S.M.S. (2006). Effect of process parameters on transmembrane flux during direct osmosis. J. Membr. Sci..

[47] Geens J., Van der Bruggen B., Vandecasteele C. (2004). Characterisation of the solvent stability of polymeric nanofiltration membranes by measurement of contact angles and swelling. Chem. Eng. Sci..

[48] Freger V., Bottino A., Capannelli G., Perry M., Gitis V., Belfer S. (2005). Characterization of novel acid-stable NF membranes before and after exposure to acid using ATR-FTIR, TEM and AFM. J. Membr. Sci..

[49] Hai Y., Zhang J., Shi C., Zhou A., Bian C., Li W. (2016). Thin film composite nanofiltration membrane prepared by the interfacial polymerization of 1,2,4,5-benzene tetracarbonyl chloride on the mixed amines cross-linked poly(ether imide) support. J. Membr. Sci..

[50] Darvishmanesh S., Degrève J., Van der Bruggen B. (2009). Comparison of pressure driven transport of ethanol/n-hexane mixtures through dense and microporous membranes. Chem. Eng. Sci..

[51] Coronell O., Mariñas B.J., Zhang X., Cahill D.G. (2008). Quantification of functional groups and modeling of their ionization behavior in the active layer of FT30 reverse osmosis membrane. Environ. Sci. Technol..

[52] Mulder M., Mulder M. (1996). Introduction. Basic Principles of Membrane Technology.

[53] Patterson D.A., Havill A., Costello S., See-Toh Y.H., Livingston A.G., Turner A. (2009). Membrane characterisation by SEM, TEM and ESEM: The implications of dry and wetted microstructure on mass transfer through integrally skinned polyimide nanofiltration membranes. Sep. Purif. Technol..

[54] Nghiem L.D., Coleman P.J., Espendiller C. (2010). Mechanisms underlying the effects of membrane fouling on the nanofiltration of trace organic contaminants. Desalination.

[55] Yang Z., Craig D.Q.M. (2020). Monitoring film coalescence from aqueous polymeric dispersions using atomic force microscopy: Surface topographic and nano-adhesion studies. Asian J. Pharm. Sci..

[56] Cadotte J.E. (1981). Interfacially Synthesized Reverse Osmosis Membrane (FilmTec FT-30). U.S. Patent.

[57] Mansourpanah Y., Madaeni S.S., Rahimpour A. (2009). Preparation and investigation of separation properties of polyethersulfone supported poly(piperazineamide) nanofiltration membrane using microwave-assisted polymerization. Sep. Purif. Technol..

[58] Xu X., Kirkpatrick R.J. (2006). NaCl interaction with interfacially polymerized polyamide films of reverse osmosis membranes: A solid-state 23Na NMR study. J. Membr. Sci..

[59] McGilvery C.M., Abellan P., Kłosowski M.M., Livingston A.G., Cabral J.T., Ramasse Q.M., Porter A.E. (2020). Nanoscale Chemical Heterogeneity in Aromatic Polyamide Membranes for Reverse Osmosis Applications. ACS Appl. Mater. Interfaces.

[60] Persson I. (1986). Solvation and complex formation in strongly solvating solvents. Pure Appl. Chem..

[61] Reichardt C., Welton T. (2010). Solvents and Solvent Effects in Organic Chemistry.

[62] Geens J., Hillen A., Bettens B., Van der Bruggen B., Vandecasteele C. (2005). Solute transport in non-aqueous nanofiltration: Effect of membrane material. J. Chem. Technol. Biotechnol..

[63] Amatore C., Carré E., Jutand A., M’Barki M.A., Meyer G. (1995). Evidence for the Ligation of Palladium(0) Complexes by Acetate Ions: Consequences on the Mechanism of Their Oxidative Addition with Phenyl Iodide and PhPd(OAc)(PPh3)2 as Intermediate in the Heck Reaction. Organometallics.

[64] Kurokhtina A.A., Larina E.V., Yarosh E.V., Lagoda N.A., Schmidt A.F. (2018). Mechanistic Study of Direct Arylation of Indole Using Differential Selectivity Measurements: Shedding Light on the Active Species and Revealing the Key Role of Electrophilic Substitution in the Catalytic Cycle. Organometallics.

[65] Iwasawa T., Tokunaga M., Obora Y., Tsuji Y. (2004). Homogeneous palladium catalyst suppressing Pd black formation in air oxidation of alcohols. J. Am. Chem. Soc..

[66] Tsuji Y., Fujihara T. (2007). Homogeneous nanosize palladium catalysts. Inorg. Chem..

[67] Nair D., Scarpello J.T., Vankelecom I.F.J., Freitas Dos Santos L.M., White L.S., Kloetzing R.J., Welton T., Livingston A.G. (2002). Increased catalytic productivity for nanofiltration-coupled Heck reactions using highly stable catalyst systems. Green Chem..

[68] Nagao Y., Kim K., Sano S., Kakegawa H., Lee W.S., Shimizu H., Shiro M., Katunuma N. (2010). ChemInform Abstract: Syntheses and Reactions of the Diethyl α-Alkynylmalonates Involving the Generation of Conjugated Allenyl Esters as the Latent Active Species: A New Approach to the Development of Cysteine Proteinase Inhibitors. ChemInform.

[69] Scarpello J.T., Nair D., Freitas Dos Santos L.M., White L.S., Livingston A.G. (2002). The separation of homogeneous organometallic catalysts using solvent resistant nanofiltration. J. Membr. Sci..

