# Supplementary material for: Characterization of Selected Polymeric Membranes Used in the Separation and Recovery of Palladium-Based Catalyst Systems
Source: Membranes (Basel). 2020 Jul 28;10(8):166. doi: 10.3390/membranes10080166 (PMC7464706; doi:10.3390/membranes10080166)
Supplement: Supplementary file 1 [file membranes-10-00166-s001.zip › Table S1 Properties of selected solvents.docx]

Table S1: Properties of selected solvents

| **Solvent** | **MW (g.mol^-1^)** | **Viscosity (cP) @ 25^o^C** | **Dielectric constant (έ) @ 20^o^C** |
| --- | --- | --- | --- |
| Water* | 18.02 | 0.89 | 78.5 |
| Acetonitrile*^+^ | 41.05 | 0.37 | 36.0 |
| Methanol* | 32.04 | 0.54 | 33.00 |
| Ethanol* | 46.07 | 1.10 | 24.60 |
| 2-Propanol*^+^ | 60.10 | 2.04 | 20.18 |

* - Solvent used for membrane characterization

^+^ - Solvent used for catalyst separation
