# Supplementary material for: Characterization of Selected Polymeric Membranes Used in the Separation and Recovery of Palladium-Based Catalyst Systems
Source: Membranes (Basel). 2020 Jul 28;10(8):166. doi: 10.3390/membranes10080166 (PMC7464706; doi:10.3390/membranes10080166)
Supplement: Supplementary file 1 [file membranes-10-00166-s001.zip › Table S2 Membrane specifications according to supplier.docx]

Table S2: Membrane specifications according to supplier

| **Membrane** | **NF270** | **NF90** | **BW30** | **XLE** |
| --- | --- | --- | --- | --- |
| Type | Aromatic  /aliphatic  polyamide | Aromatic polyamide | | |
| Molecular Weight Cut-off (Da) | 150 | 200 | N/A | N/A |
| Pressure (Bar) | 41 | 41 | 41 | 41 |
| pH range | 3-10 | 3-10 | 2-11 | 2-11 |
| Permeate flow at std test conditions (l.h^-1^) | 83-1620 | 134-2300 | 120-1660 | 52-2040 |
