# Supplementary material for: Characterization of Selected Polymeric Membranes Used in the Separation and Recovery of Palladium-Based Catalyst Systems
Source: Membranes (Basel). 2020 Jul 28;10(8):166. doi: 10.3390/membranes10080166 (PMC7464706; doi:10.3390/membranes10080166)
Supplement: Supplementary file 1 [file membranes-10-00166-s001.zip › Table S3 Results of MWCO measurements.docx]

Table S3: Results of MWCO measurements

| **Membrane** | **MWCO** (Da) |
| --- | --- |
| NF90 | 140 |
| NF270 | 205 |
| BW30 | 120 |
| XLE | 100 |
