# Supplementary material for: Characterization of Selected Polymeric Membranes Used in the Separation and Recovery of Palladium-Based Catalyst Systems
Source: Membranes (Basel). 2020 Jul 28;10(8):166. doi: 10.3390/membranes10080166 (PMC7464706; doi:10.3390/membranes10080166)
Supplement: Supplementary file 1 [file membranes-10-00166-s001.zip › Table S4 Key functional group peaks.docx]

**Table S4**: Key functional group peaks

| **Membranes** | **Key Peaks (cm^-1^)** |
| --- | --- |
| NF90 | 1663.6, 1584.4, 1542.6, 1486.8, and 1238.1 |
| NF270 | 1584.9, 1543.9, 1487.4, and 1238.8 |
| BW30 | 1663.0, 1583.9, 1542.3, 1503.3, 1486.3, and 1235.9 |
| XLE | 1663.5, 1585.1, 1543.6, 1487.3, and 1238.6 |
