# Supplementary figures and images for: Characterization of Selected Polymeric Membranes Used in the Separation and Recovery of Palladium-Based Catalyst Systems
Source: Membranes (Basel). 2020 Jul 28;10(8):166. doi: 10.3390/membranes10080166 (PMC7464706; doi:10.3390/membranes10080166)

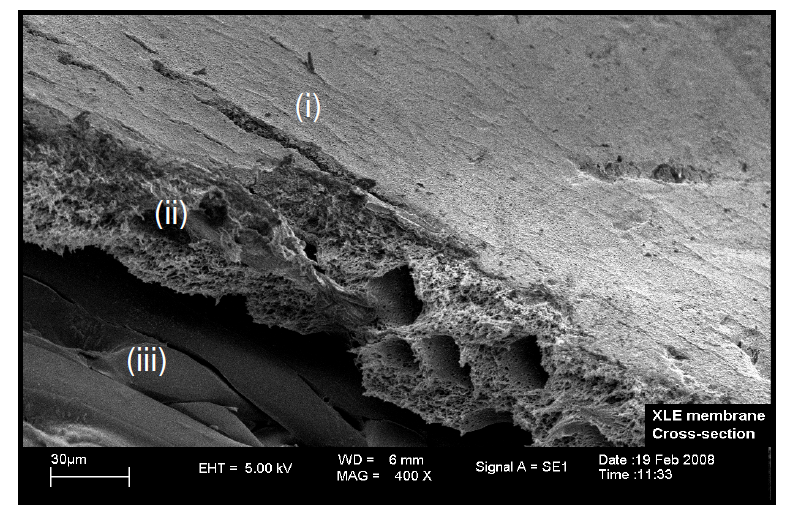


**Figure S1**: SEM micrograph showing the cross-sectional view of XLE membrane.

Supplement: Supplementary file 1 [file membranes-10-00166-s001.zip › Figure S1 SEM micrograph showing the cross-sectional view of XLE membrane..docx]

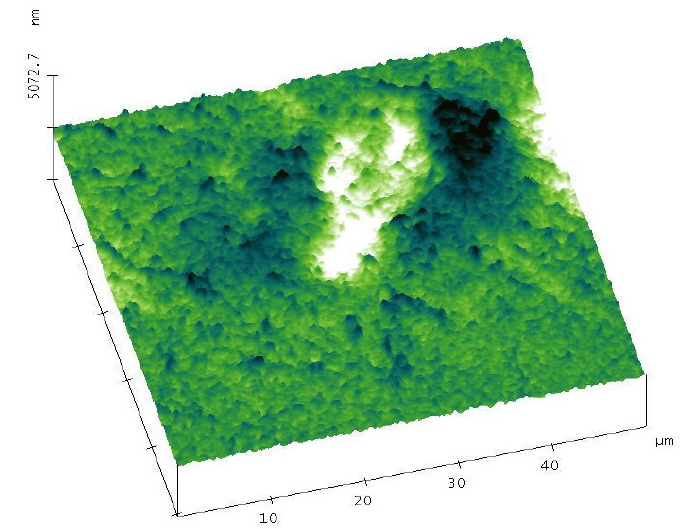


**Figure S2:** AFM image of NF90 showing characteristic surface detail

Supplement: Supplementary file 1 [file membranes-10-00166-s001.zip › Figure S2 AFM image of NF90 showing characteristic surface detail.docx]
